# Inhibition of Bruton's Tyrosine Kinase Alleviates Monocrotaline-Induced Pulmonary Arterial Hypertension by Modulating Macrophage Polarization

**DOI:** 10.1155/2022/6526036

**Published:** 2022-08-29

**Authors:** Min Yu, Xuecheng Wu, Liyao Peng, Mingxia Yang, Hong Zhou, Jian Xu, Jingjing Wang, Hong Wang, Weiping Xie, Hui Kong

**Affiliations:** ^1^Department of Respiratory and Critical Care Medicine, The First Affiliated Hospital of Nanjing Medical University, Nanjing, Jiangsu 210029, China; ^2^Department of Respiratory and Critical Care Medicine, The Affiliated Suzhou Hospital of Nanjing Medical University, Suzhou Municipal Hospital, Suzhou, Jiangsu 215000, China; ^3^Department of Respiratory and Critical Care Medicine, The Affiliated Changzhou No. 2 People's Hospital of Nanjing Medical University, Changzhou 213003, China; ^4^Department of Respiratory Medicine, Shanghai Pulmonary Hospital, Tongji University School of Medicine, Shanghai 200433, China

## Abstract

Macrophage accumulation and activation contribute to the development of pulmonary arterial hypertension (PAH), while Bruton's tyrosine kinase (BTK) is an important regulator for the activation and polarization of macrophage. However, the role of BTK in PAH remains unknown. In the present study, a selective BTK inhibitor (BTKi) BGB-3111 was applied to investigate the role of BTK in monocrotaline- (MCT-) induced PAH rat and phorbol myristate acetate- (PMA-) differentiated U937 macrophages. Our results showed that BTK was mainly distributed and upregulated in CD68^+^ macrophages in the lungs of PAH rats. Daily treated with BTKi BGB-3111 alleviated MCT-induced PAH, as indicated by the decrease in right ventricular systolic pressure (RVSP), attenuation in right ventricle hypertrophy and pulmonary vascular remodeling, reduction in perivascular collagen deposition, as well as inhibition of inflammation and endothelial-to-mesenchymal transition (EndMT) in the lung. Moreover, BTK inhibition suppressed MCT-induced recruitment of macrophages, especially the classical activated macrophages (M1) in the lung. *In vitro*, BGB-3111 significantly suppressed lipopolysaccharide- (LPS-) induced M1 polarization and proinflammatory cytokine production in U937-derived macrophages. The underlying mechanism is associated with the inhibition of NF-*κ*B/MAPK pathways and nucleotide-binding oligomerization domain-like receptor with pyrin domain 3 (NLRP3) inflammasome activation. Furthermore, macrophage conditioned medium (CM) from LPS-induced M1 macrophages promoted migration and EndMT of HPAECs, while CM from BGB-3111-pretreated LPS-induced M1 macrophages failed to induce this response. These findings suggest that BTK inhibition alleviates PAH by regulating macrophage recruitment and polarization and may be a potential therapeutic strategy for the treatment of PAH.

## 1. Introduction

Pulmonary arterial hypertension (PAH) is a devastating pulmonary vasculopathy characterized by pulmonary vasoconstriction and vascular remodeling, ultimately leading to right heart failure and death [1]. Pathogenesis of PAH includes pulmonary vascular endothelial dysfunction, pulmonary artery smooth muscle cell proliferation and migration, medial hypertrophy, inflammation, vasoconstriction, and thrombosis *in situ* [2]. Although current therapeutic approaches targeted on three major well-characterized pathogenic pathways (including nitric oxide, endothelin, and prostacyclin) have achieved great success for the treatment of PAH, the long-term survival of PAH remains dismal [3]. Therefore, new targets and therapeutics are urgently needed for improving the prognosis and survival of patients with PAH.

In recent years, increasing attention has been focused on the effects of perivascular inflammation in the development of PAH [4]. In both experimental and clinical PAH, CD68^+^ macrophages are the predominant inflammatory cells accumulating in the adventitia of distal pulmonary arteries and alveolar septa [5, 6]. Inactivation or depletion of macrophages in lung prevents hypoxia-induced pulmonary hypertension and portopulmonary hypertension [7–9]. Mounting evidence demonstrates that macrophages retain considerable plasticity to alter their effector phenotypes [10]. Undifferentiated M0 macrophages can polarize into classical proinflammatory M1 or anti-inflammatory M2 macrophages in response to microenvironmental changes. Inflammatory stimuli such as lipopolysaccharide (LPS) or IFN-*γ* induce macrophage M1 polarization, characterized by the expression of high levels of CD80, CD86, inducible nitric oxide synthase (iNOS), and chemokines (such as CXCL1, CXCL3, and CXCL10). Activated M1 macrophages produce a battery of proinflammatory cytokines including interleukin-6 (IL-6), IL-1*β*, and tumor necrosis factor-*α* (TNF-*α*). In contrast, anti-inflammatory M2 macrophages, which can be induced by IL-4 or IL-10, are characterized by the expression of CD163, mannose receptor, arginase-1 (Arg-1), IL-10, and chemokines (CCL2, CCL17, and CCL22). Macrophage plasticity or phenotypic heterogeneity has proved useful for understanding the complexity of several diseases, including PAH [11]. Recently, it was reported that modulation of macrophage recruitment and/or phenotype can be a novel therapeutic approach for PAH [6]. Thus, further research is needed to identify the genetic and epigenetic mechanisms of macrophage heterogeneity and polarization.

Bruton's tyrosine kinase (BTK) is a nonreceptor tyrosine kinase of the Tec family contributing to the differentiation and activation of B cells. Moreover, BTK also plays an important role in B cell malignancies, inflammatory autoimmune conditions (including rheumatoid arthritis, systemic lupus erythematosus, multiple sclerosis, systemic sclerosis, and Sjögren's syndrome), liver damage, thromboembolism, and ischaemic brain injury [12–15]. In the context of hypoxic inflammation, BTK activation mediated macrophage recruitment and proliferation [16]. Pharmacologic or genetic inhibition of BTK suppressed the production of proinflammatory cytokines by macrophages induced by immune-complex or LPS, suggesting that BTK is an important regulator for the activation and polarization of macrophages [17, 18].

Considering the effects of macrophage in the development of PAH, we hypothesized that BTK might involve the development of PAH by regulating macrophage-mediated inflammation. To test this hypothesis, a novel and selective BTK inhibitor (BTKi), BGB-3111, was employed to explore the role of BTK in monocrotaline- (MCT-) induced PAH in rats. Moreover, the effects and mechanisms of BTKi on macrophage M1/M2 polarization were investigated both *in vivo* and *in vitro*.

## 2. Material and Methods

### 2.1. Ethical Approval

All animal care and experimental procedures were approved by the Institutional Animal Care and Use Committee of Nanjing Medical University (NJMU/IACUC-1809008) and were performed according to the Guide for the Care and Use of Laboratory Animals published by the National Institutes of Health (NIH Publication No. 85-23, revised 1996).

### 2.2. Experimental Animals and Design

Male adult Sprague-Dawley rats (200-220 g, Nanjing Medical University Experimental Animal Center, Nanjing, China) were kept under standard conditions with free access to water and food for one week. The PAH rat model was established by a single intraperitoneal injection of MCT (60 mg/kg, Sigma-Aldrich, St. Louis, MO), while the control groups were injected with normal saline only. To establish a model of PAH, rats were randomly assigned into two groups as follows: (1) control group (*n* = 6), (2) MCT group (*n* = 6). To analyze the effects of BTKi in MCT-induced PAH, rats were randomly assigned into four groups as follows: (1) control group (*n* = 8), (2) BGB-3111 group (160 mg/kg/day, *n* = 8), (3) MCT group (*n* = 8), (4) MCT + BGB-3111 group (40, 80, and 160 mg/kg/day, *n* = 8 at each dose). BGB-3111 was provided by BeiGene (Beijing, PR China) and was administered intragastrically daily for 3 weeks starting 4 h before MCT injection. Methyl cellulose (10%) was used as a vehicle. Hemodynamic measurement and histological analysis were both evaluated at day 21.

### 2.3. Hemodynamic Measurement and Specimen Collection

At the end of the experiment, all rats were anesthetized with an intraperitoneal injection of sodium pentobarbital (45 mg/kg) before right-heart catheterization. The right ventricular systolic pressure (RVSP) was recorded using a PowerLab data acquisition system (ADI Instruments). Bronchoalveolar lavage fluid (BALF) was obtained in the lung through an intratracheal cannula with saline. Macrophages in BALF were counted using a hemocytometer immediately. Then, the right lung tissues were flushed with cold saline through the pulmonary artery and were excised for protein isolation and histological assessment. The weight ratio of the right ventricle to the left ventricle plus the interventricular septum [RV/(LV + S)] was calculated as the right ventricular hypertrophy index.

### 2.4. Histological Analysis

Lung tissue samples were fixed in 4% paraformaldehyde, embedded in paraffin, and sectioned at 5 *μ*m intervals. Hematoxylin-eosin (H&E), Elastica van Gieson (EVG), and Masson trichrome staining (MTS) were performed using standard procedures. The sections were then examined and photographed with a Leica 2500 microscope (Leica Microsystems, Wetzlar, Germany). The pulmonary arterial wall thickness was calculated as follows: medial wall thickness (%) = (external diameter–internal diameter)/external diameter × 100. For quantitative analysis, 20 randomly selected vessels with an external diameter of 25–100 *μ*m from each rat were analyzed.

### 2.5. Immunohistochemistry and Immunofluorescence Analysis

To assess the muscularization of small pulmonary arteries, immunohistochemical staining was performed with an antibody against *α*-smooth muscle actin (*α*-SMA). The infiltration of macrophages was evaluated by CD68 immunohistochemistry method. Briefly, lung sections were deparaffinized followed by antigen retrieval and endogenous peroxidase removal. After 1-hour blockage with 5% BSA, the sections were incubated with primary antibodies of *α*-SMA (1 : 100, Abcam, Cambridge, MA, USA) and CD68 (1 : 100, Abcam) overnight at 4°C and HRP-conjugated secondary antibody (1 : 1000, Proteintech) for 1 h at room temperature. The reactions were visualized using diaminobenzidine chromogenic solution. The degree of muscularization was defined by the proportion of *α*-SMA-positive parts of circumference according to a previous study [19]. The muscularization of distal vessels with diameter 25–100 *μ*m was quantified, and the percentages of nonmuscularized, partially muscularized, and fully muscularized vessels were calculated.

For double immunofluorescence staining, tissue sections were performed as previously described [20]. To examine the expression and distribution of BTK in lung tissues, the sections were incubated overnight at 4°C in a combination of anti-CD31, anti-*α*-SMA, anti-CD68, anti-CD45, and anti-BTK (1 : 100, Abcam). Sections were then labeled with the second antibody (donkey anti-mouse IgG, Alexa Fluor 555; and donkey anti-rat IgG, Alexa Fluor 488) for 1 h at room temperature. Nuclei were stained with 4, 6-diamidino-2-phenylindole (DAPI, Sigma-Aldrich). Fluorescence images were also captured by the use of the Leica 2500 microscope.

### 2.6. Cell Culture

Human monocytic leukemia U937 cells were cultured at 37°C in a humidified atmosphere with 5% CO_2_ in RPMI-1640 medium supplemented with 10% heat-inactivated fetal bovine serum and 1% penicillin/streptomycin solution. According to previous studies, U937 monocytes were treated with 100 ng/ml phorbol myristate acetate (PMA) for 48 h to induce cellular differentiation into unpolarized macrophages (M0) [21]. To establish the M1 polarization of macrophages, the M0 macrophages were stimulated with 100 ng/ml LPS (Sigma-Aldrich) for an additional 24 h. Macrophages were treated with varying concentrations of BGB-3111 at the same time.

Human pulmonary arterial endothelial cells (HPAECs), purchased from ScienCell Research Laboratories, were maintained in endothelial cell medium (ScienCell) with 5% fetal bovine serum, 1% endothelial cell growth supplement, and 1% penicillin/streptomycin solution at 5% CO_2_ and 37°C. Macrophage conditioned medium (CM) was mixed with endothelial cell medium at a ratio of 1 : 2. For detecting endothelial-to-mesenchymal transition (EndMT), cells were stimulated by CM for 48 h, while for Transwell migration assays, cells were challenged by CM for 24 h.

### 2.7. Preparation of Macrophage Conditioned Medium

U937 cells were treated with PMA for 48 h to induce monocyte-macrophage differentiation. After that, U937-derived macrophages were washed and then treated with the following additives to their culture medium: (1) no additional additive to maintain M0 macrophages; (2) 100 ng/ml LPS to induced M1 macrophages polarization; (3) 40 *μ*M BGB-3111; and (4) 100 ng/ml LPS+40 *μ*M BGB-3111. After incubation for 24 h, the medium was removed, and all cells were washed 3 times with phosphate-buffered saline (PBS) to remove residual LPS and other additives, and then, the cells were cultured in fresh RPMI-1640 medium for 8 h. Supernatant conditioned medium was collected from each cell group and labeled as follows: (1) Con-CM, (2) LPS-CM, (3) BGB-3111-CM, (4) LPS+BGB-3111-CM. Next, the supernatants of these cultures were collected and centrifuged at 1000 × g at 4°C for 10 min and then stored at –80°C until cytokine profile analysis.

### 2.8. Flow Cytometry for Macrophage Subtype Analysis

Phenotypic analysis of the macrophages was performed using flow cytometry. Cells in bronchoalveolar lavages were resuspended in 0.9% sterile saline and incubated with an Fc receptor blocker (BD Biosciences) for 15 min at 4°C to reduce nonspecific binding. Next, cells were stained with a mixture of anti-CD68-FITC, anti-CD86-PE, and anti-CD163-Alexa Fluor 647 antibodies (AbD Serotec, Kidlington, UK) for 30 min at 4°C. Subsequently, cells were washed with PBS, and flow cytometry analysis was carried out using a BD FACSCalibur Flow Cytometer.

U937-derived macrophages were pretreated with or without BGB-3111 and then costimulated with or without LPS (100 ng/ml) for 24 h. The cells were collected and washed 3 times with ice-cold PBS. Firstly, the cells were incubated with ice-cold PBS containing 5% serum at 4°C to avoid nonspecific binding. After the treatment, the cells were collected and stained for anti-human-CD86-FITC antibodies (BD pharmingen, San Diego, CA, USA) for 30 min at 4°C in the dark. After immunostaining, the cells were washed twice with PBS and analyzed using the BD FACSCalibur Flow Cytometer. Results were calculated using FlowJo software (Tree Star Inc., Ashland, OR).

### 2.9. Cell Migration Assay

Transwell migration assay was conducted using a 24-well Transwell chamber (Corning, Corning, NY, USA) to examine the migration of HPAECs in different conditioned media. HPAECs (5 × 10^4^ cells) were loaded into the upper chamber, and different macrophage conditioned media were added to the lower chamber. After 24 h, the upper surface of the membrane was scraped using a cotton swab and cells on the lower surface were fixed in 4% paraformaldehyde for 30 min and stained with 5% crystal violet (Beyotime, China) for 30 min. For each filter, at least five randomly chosen fields were imaged using a phase contrast microscope (Nikon, Tokyo, Japan).

### 2.10. Enzyme-Linked Immunosorbent Assay (ELISA)

Cytokine profile in rat lung tissues and conditioned medium from U937-derived macrophages were measured by ELISA. The concentration of IL-6, IL-1*β*, and TNF-*α* was determined using enzyme immunoassay kits (Cusabio Biotech Co., Ltd., Wuhan, China) according to the manufacturer's instructions.

### 2.11. Quantitative Real-Time PCR (RT-qPCR)

Total RNA was extracted from lung tissues or cultured cells using the RNAiso plus (Takara, Japan). Reverse transcription reactions were performed with 500 ng of total RNA and a PrimeScript™ RT Reagent Kit (TaKaRa, Japan) according to the manufacturer's protocol. RT-qPCR was performed using the ABI Prism 7500 FAST apparatus (Applied Biosystems, Foster City, CA, USA). The primer sequences are shown in Table 1. The 2^–ΔΔCt^ method was used to quantify mRNA expression relative to *β*-actin.

### 2.12. Western Blotting Analysis

Western blotting was performed as previously described [20]. Briefly, protein extracted from cells or isolated lung tissues were electrophoresed through 10% SDS-PAGE, transferred to a PVDF membrane, and incubated with primary antibodies against BTK, iNOS, Arg-1, vascular endothelial cadherin (VE-cadherin), *α*-SMA, Vimentin and Fibronectin (1 : 1000, Abcam), nucleotide-binding oligomerization domain-like receptor with pyrin domain 3 (NLRP3) and Caspase-1 (1 : 1000, Santa Cruz, CA, USA), p-p38/p38 MAPK, p-ERK1/2/ERK1/2, p-I*κ*B*α*/I*κ*B*α*, and NF-*κ*B p-p65/p65 (1 : 1000, Cell Signaling Technology, Danvers, MA), and *β*-actin (1 : 5000, Proteintech). The membranes were then incubated with HRP-conjugated secondary antibodies, and bands were visualized using the WesternBright ECL reagent (Advansta, CA). Densitometric quantification was performed using Image J software.

### 2.13. Statistical Analysis

Data were analyzed in SPSS 18.0 software (SPSS Inc., Chicago, IL, USA) by Student's *t*-tests or one-way ANOVA followed by LSD post hoc test. All data were expressed as mean ± standard error of the mean. *P* values < 0.05 was considered statistically significant.

## 3. Results

### 3.1. BTK Expression Was Increased in the Lungs of MCT-Induced PAH Rats

**Figure 1:**
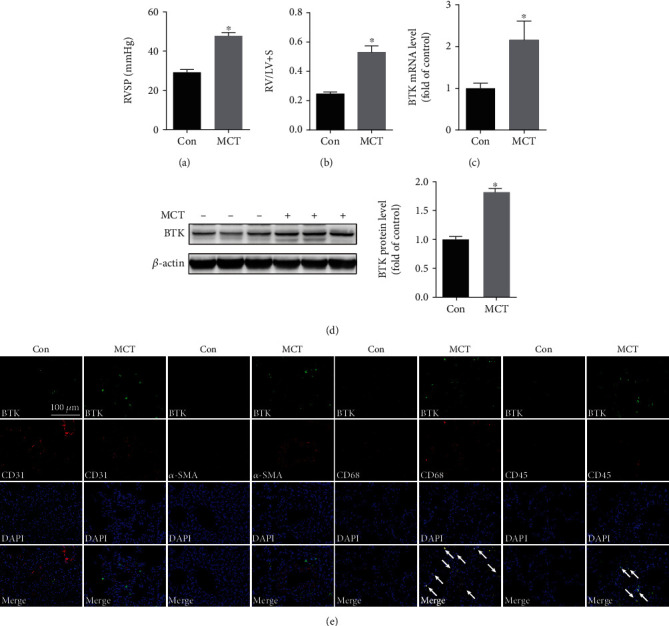
Expression of BTK (Bruton's tyrosine kinase) was increased in rats with monocrotaline- (MCT-) induced pulmonary arterial hypertension (PAH). (a, b) Measurement of right ventricular systolic pressure (RVSP) and weight ratio of right ventricular to the left ventricle and interventricular septum [RV/(LV + S)] in control group rats and MCT group rats. (c) BTK mRNA level was measured by RT-qPCR. (d) BTK protein level was measured by Western blot. (e) Representative immunofluorescence images of lung sections stained for BTK with CD31, *α*-SMA, CD68, or CD45 from control and MCT-treated rats. Data are presented as mean ± SEM, *n* = 6. ^∗^*P* < 0.05 vs. control.

MCT-induced PAH rat model was employed to examine the expression and distribution of BTK in lung tissues. Hemodynamics analyses and right ventricular hypertrophy index [RV/(LV + S)] verified the successful establishment of PAH (Figures 1(a) and 1(b)). RT-qPCR and Western blotting analyses showed that both mRNA and protein level of BTK in the lung of MCT-induced PAH were significantly elevated than those in the control group (Figures 1(c) and 1(d)). Double immunofluorescence staining showed that BTK was upregulated and mainly colocalized with macrophages (CD68^+^), rather than smooth muscle cells (*α*-SMA^+^) or endothelial cells (CD31^+^) (Figure 1(e)). In addition, only a few BTK positive cells colocalized with leukocyte (CD45^+^). These results indicated that BTK was significantly upregulated and dominantly expressed in macrophages in lungs of MCT-induced PAH rats.

### 3.2. BTK Inhibition Attenuated MCT-Induced PAH, Pulmonary Vascular Remodeling, and Endothelial-to-Mesenchymal Transition In Vivo

**Figure 2:**
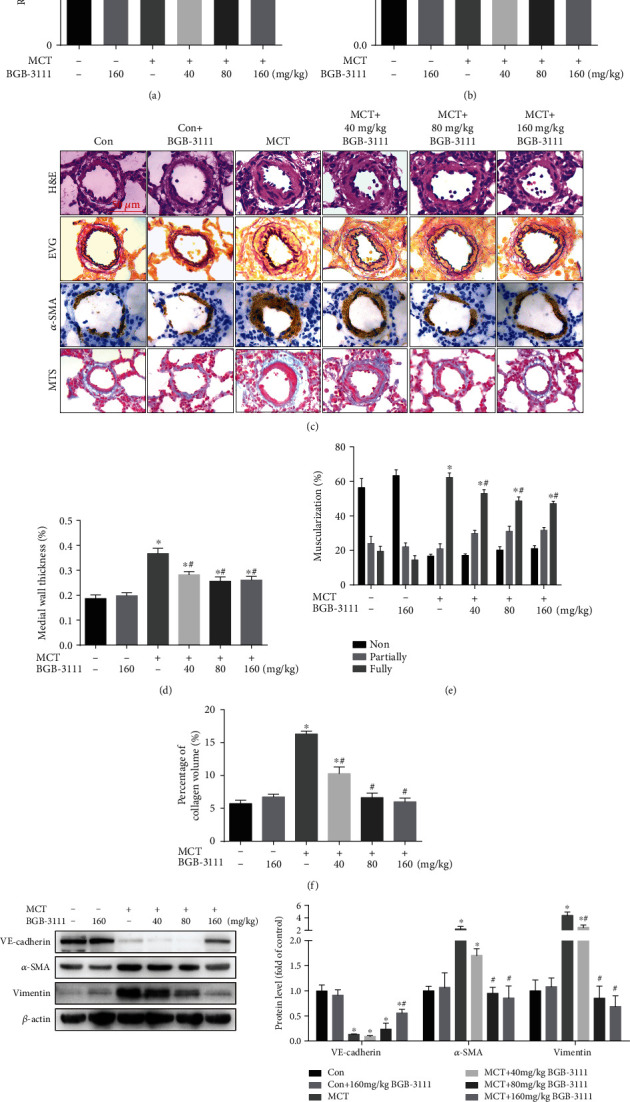
BTK inhibition attenuated MCT-induced PAH, pulmonary vascular remodeling, collagen deposition, and endothelial-to-mesenchymal transition. (a, b) Measurement of the RVSP and [RV/(LV + S)] in each treatment group. (c) Representative histological images of H&E staining, EVG staining, immunohistochemistry for *α*-SMA, and Masson trichrome staining (MTS) in pulmonary arteries (external diameter, 25–100 *μ*m). (d) Quantifications of medial wall thickness of pulmonary arteries in different groups. (e) Quantification of pulmonary vascular muscularization determined by *α*-SMA staining. Nonmuscularized vessels (Non), partially muscularized vessels (Partially), and fully muscularized vessels (Fully) were analyzed. (f) Pulmonary vascular fibrosis in each treatment group calculated by Masson trichrome staining (*n* = 6–8 for each group). (g) Representative images and analysis of blotting for VE-cadherin, *α*-SMA, and Vimentin in rat lung tissues (*n* = 3–5). Data are presented as mean ± SEM. ^∗^*P* < 0.05 vs. control; ^#^*P* < 0.05 vs. MCT.

To investigate the effects of BTK in MCT-induced PAH, BGB-3111, a selective BTK inhibitor, was administrated to rats starting 4 h before MCT injection. Hemodynamic data showed that RVSP in MCT-treated rats was significantly higher than those in control rats, while BGB-3111 treatment markedly decreased MCT-induced elevation of RVSP (Figure 2(a)). Meanwhile, BGB-3111 dose-dependently inhibited MCT-induced right ventricular hypertrophy (Figure 2(b)). Histological analysis by H&E and EVG staining showed that pulmonary artery medial wall thickness in rats treated with MCT was significantly elevated compared to control rats, which was attenuated by BGB-3111 (Figures 2(c) and 2(d)). Muscularization of the small pulmonary arteries (external diameter, 25-100 *μ*m) was determined by *α*-SMA immunostaining. BGB-3111 significantly reduced MCT-induced muscularization of small pulmonary arteries (Figures 2(c) and 2(e)). Pulmonary vascular adventitia fibrosis was evaluated by Masson trichrome staining (MTS). Furthermore, BGB-3111 treatment partially attenuated collagen deposition in the distal pulmonary arteries in rats with MCT-induced PAH (Figures 2(c) and 2(f)). In nowadays, EndMT has emerged as a pivotal mechanism not only in the pathogenesis of fibrosis but also in the pulmonary vascular remodeling processes in pulmonary hypertension. Thus, we investigated the effect of BGB-3111 on the EndMT-related markers in the lung. Western blot analysis indicated that BGB-3111 partly restored MCT-induced downregulation of endothelial marker VE-cadherin, while suppressed MCT-induced upregulation of mesenchymal markers *α*-SMA and Vimentin (Figure 2(g)) in lung tissue. We then measured the expression of BTK in the lung. As indicated in Supplementary Figure 1, BGB-3111 suppressed MCT-induced upregulation of BTK in lung tissue. The above results indicated that inhibition of BTK can alleviate MCT-induced PAH, pulmonary vascular remodeling, right ventricle hypertrophy, pulmonary vascular fibrosis, and EndMT.

### 3.3. BTK Inhibition Attenuated MCT-Induced Macrophage-Related Inflammation in the Lung

**Figure 3:**
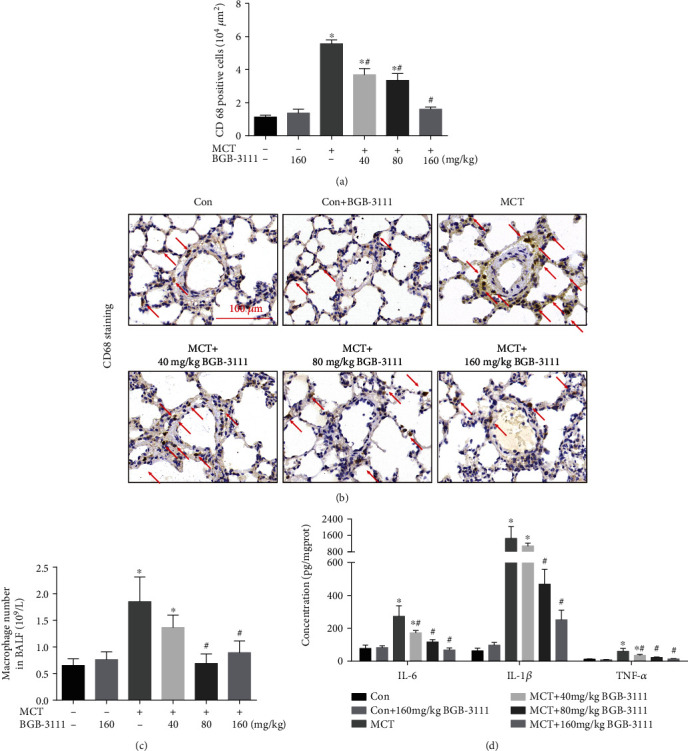
BTK inhibition attenuated MCT-induced macrophage-related inflammation in the lung. (a) Quantification of CD68^+^ macrophages in rat lung tissues. (b) Representative images of CD68 immunohistochemistry in each group. (c) Macrophage count in bronchoalveolar lavage of each group. (d) The expression levels of proinflammatory cytokines IL-6, IL-1*β*, and TNF-*α* in lung were measured by ELISA. Data are presented as mean ± SEM, *n* = 6–8. ^∗^*P* < 0.05 vs. control; ^#^*P* < 0.05 vs. MCT.

Inflammation and inflammatory cell infiltration (especially macrophages) have been found to exacerbate pulmonary vascular remodeling in the development of PAH [22]. To examine the effect of BTKi on MCT-induced pulmonary inflammation, macrophage infiltration and macrophage related proinflammatory cytokines (IL-6, IL-1*β*, and TNF-*α*) in lung tissue were detected. Macrophage infiltration was determined by immunohistochemical staining of CD68 in lung tissue and cell counting in BALF. Immunohistochemical assay of CD68 revealed that the number of perivascular macrophages was markedly increased in MCT-induced PAH rats. However, it was significantly alleviated by BGB-3111 dose-dependently (Figures 3(a) and 3(b)). In addition, BGB-3111 treatment inhibited MCT-induced macrophage recruitment in BALF (Figure 3(c)). ELISA data showed that inhibiting BTK by BGB-3111 dose-dependently suppressed MCT-induced upregulation of IL-6, IL-1*β*, and TNF-*α* in the lung (Figure 3(d)).

### 3.4. BTK Inhibition Regulated Distribution of Polarized Macrophage Subsets in the Lung in MCT-Induced PAH

**Figure 4:**
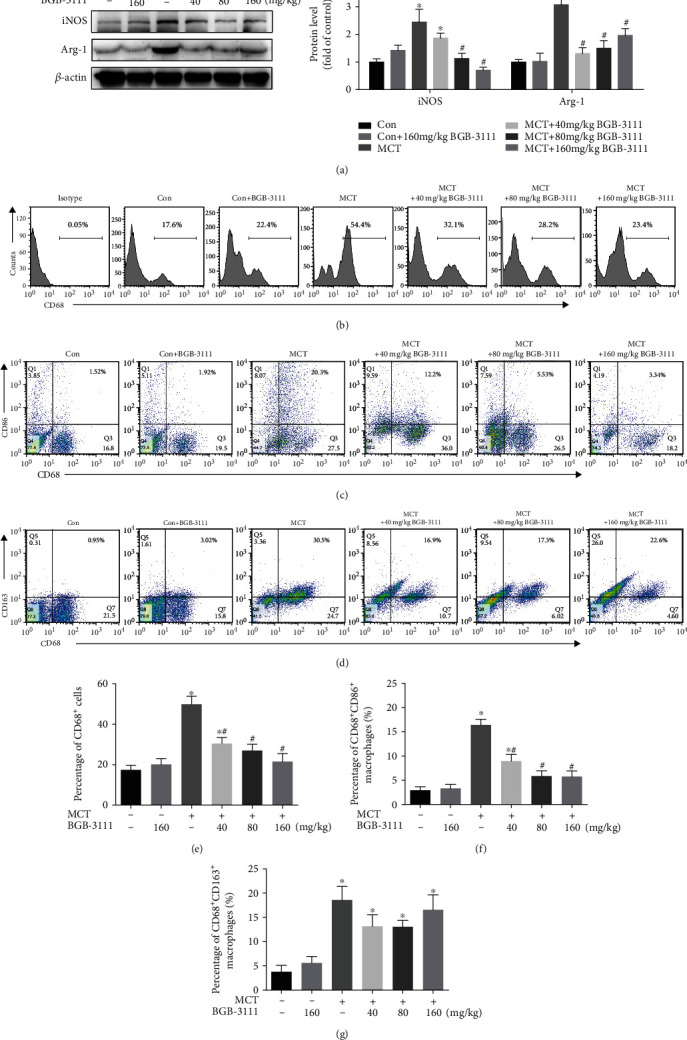
BTK inhibition regulated macrophage polarization in MCT-induced PAH. (a) The expression levels of iNOS and Arg-1 in rat lung tissues were measured by Western blot (*n* = 3–5). Expression of CD68^+^ (macrophages), CD68^+^CD86^+^ (M1), and CD68^+^CD163^+^ (M2) in bronchoalveolar lavage was evaluated by flow cytometry analysis. (b–d) Representative images of flow cytometry analysis. (e–g) Percentages of CD68^+^ macrophages, CD68^+^CD86^+^ macrophages, and CD68^+^CD163^+^ macrophages in bronchoalveolar lavage of each treatment group (*n* = 6–8). Data are presented as mean ± SEM. ^∗^*P* < 0.05 vs. control; ^#^*P* < 0.05 vs. MCT.

Since macrophage polarization plays a critical role in the development and progression of pulmonary hypertension [23], expressions of iNOS and Arg-1 and the marker of M1 and M2 macrophage, respectively, in the lung were determined by Western blot. Our data showed that MCT enhanced the expression of both iNOS and Arg-1 in the lung, which was inhibited by the treatment of BGB-3111 (Figure 4(a)). Further, macrophage polarization was determined by flow cytometry analysis of M1 and M2 macrophages in BALF. Compared to the control group, MCT administration resulted in intensive recruitment of CD68^+^ macrophages in BALF (Figures 4(b) and 4(e)), which was dose-dependently inhibited by the treatment of BGB-3111. Moreover, cytometry analysis of M1 macrophages (CD68^+^/CD86^+^) and M2 macrophages (CD68^+^/CD163^+^) indicated that MCT administration led to dramatic recruitment of both M1 and M2 macrophages in BALF. Interestingly, BGB-3111 treatment significantly suppressed MCT-induced M1 macrophages recruitment rather than M2 macrophages recruitment in BALF (Figures 4(c), 4(d), 4(f), and 4(g)).

NF-*κ*B and MAPK pathways have been reported to be associated with various forms of PAH [24, 25]. To further explore the underlying mechanisms of BGB-3111 in PAH, we examined the effects of BGB-3111 on MAPK and NF-*κ*B signaling pathways in lung tissue. As shown in Supplementary Figure 2, MCT exposure triggered the phosphorylation of p65 NF-*κ*B, ERK1/2, and p38 MAPK in the lung tissue of rats, all of which were significantly attenuated in both the 80 and 160 mg/kg/day BGB-3111-treated groups. These results indicated that inhibition of BTK can alleviate MCT-induced PAH at least partly via inhibition of the MAPK and NF-*κ*B pathways.

### 3.5. BTK Inhibition Reduced LPS-Induced M1 Macrophage Polarization In Vitro

**Figure 5:**
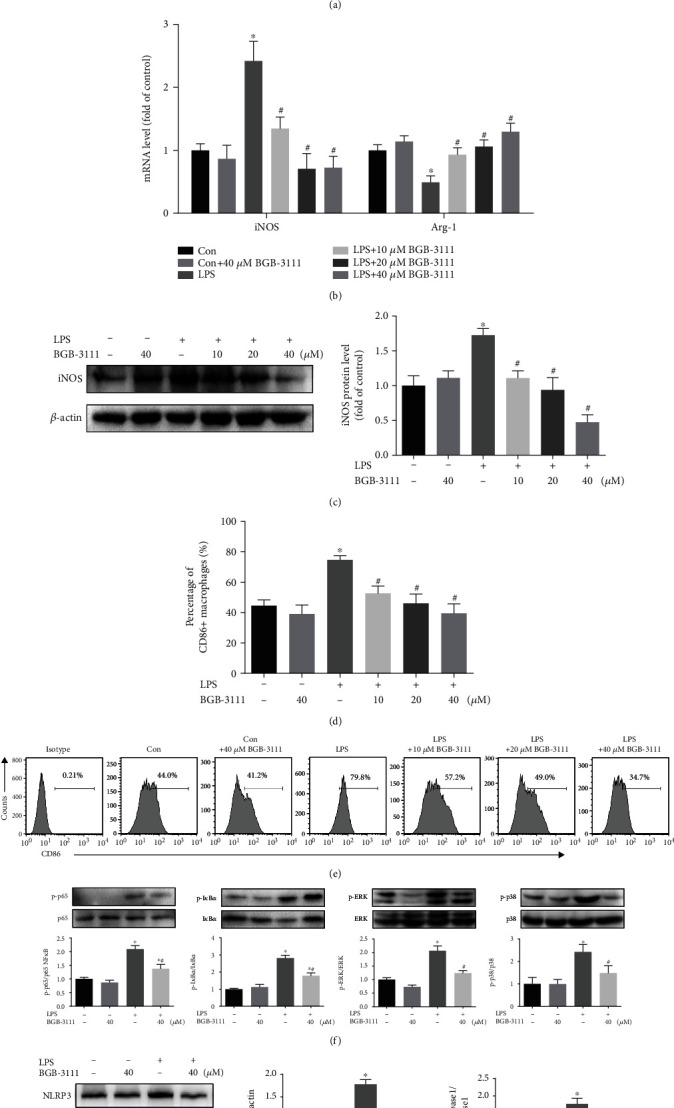
BTK inhibition reduced LPS-induced M1 macrophage polarization *in vitro*. U937-derived macrophages were exposed to the indicated conditions for 24 h and then harvested for RNA extraction. (a, b) The mRNA levels of proinflammatory cytokines (IL-6, IL-1*β*, and TNF-*α*) and macrophage-specific enzyme (iNOS and Arg-1) were measured by RT-qPCR in each treatment group (*n* = 5–6). (c) The expression of iNOS in macrophages was measured by Western blot (*n* = 3). Expression of CD86 (M1) in U937-derived macrophages was evaluated by flow cytometry analysis. (d) Percentages of CD86^+^ M1 macrophages (*n* = 5). (e) Representative FACS plots are shown. (f) Western blots were used to determine the phosphorylation of p65 NF-*κ*B, I*κ*B*α*, ERK, and p38 MAPK in macrophages (*n* = 4). (g) The expression of NLRP3 and procaspase-1 and cleaved caspase-1 in macrophages was measured by Western blot (*n* = 3–4). Data are presented as mean ± SEM. ^∗^*P* < 0.05 vs. the control group; ^#^*P* < 0.05 vs. the LPS group.

Our *in vivo* results indicate that BTK inhibition suppresses recruitment of interstitial and alveolar macrophages in the lung. It has been reported that interstitial macrophages are derived from blood monocytes, and alveolar macrophages are renewed by monocytes with interstitial macrophages as intermediates [26, 27]. To further determine whether BTK could regulate M1 macrophage polarization directly *in vitro*, U937 monocyte-derived macrophages were stimulated by LPS with or without BGB-3111. Our results showed that BGB-3111 dose-dependently suppressed LPS-induced mRNA transcription of M1-related proinflammatory cytokines (IL-6, IL-1*β*, and TNF-*α*) (Figure 5(a)). Moreover, BGB-3111 significantly inhibited LPS-induced upregulation of M1-specific enzyme iNOS mRNA and restored downregulation of M2-related molecule Arg-1 mRNA in macrophages (Figure 5(b)). Western blot analysis further confirmed that LPS enhanced the protein level of iNOS, which was reversed by BGB-3111 (Figure 5(c)). Then, the effects of BGB-3111 on macrophage polarization were investigated by flow cytometry analysis (Figures 5(d) and 5(e)). M1 macrophages were identified as viable CD86^+^ cells. Flow cytometry analysis showed that BGB-3111 effectively reduced the proportion of M1 macrophages in response to LPS. Taken together, these results demonstrated that BGB-3111 could inhibit LPS-induced macrophage M1 polarization.

Activation of NF-*κ*B and MAPK signaling pathways is crucial for M1 polarization and associated with proinflammatory cytokine production induced by LPS [28]. Our results showed that LPS challenge triggered significant phosphorylation of ERK1/2, p38 MAPK, NF-*κ*B p65, and I*κ*B*α* in U937-derived macrophages (Figure 5(f)). However, pretreated with BGB-3111 significantly inhibited LPS-induced phosphorylation of p65 NF-*κ*B and I*κ*B*α* and completely suppressed LPS-induced phosphorylation of ERK1/2 and p38 MAPK. NLRP3 inflammasome is a key platform for the maturation of IL-1*β* in macrophage. Therefore, the effects of BGB-3111 on the expression of NLRP3 and downstream effector Caspase-1 in U937-derived macrophages were investigated. BGB-3111 treatment also blocked LPS-induced upregulation of NLRP3 and the cleavage of pro-caspase-1 (Figure 5(g)).

### 3.6. BTK Inhibition Protected Endothelial-to-Mesenchymal Transition In Vitro by a Direct Effect on Macrophages

**Figure 6:**
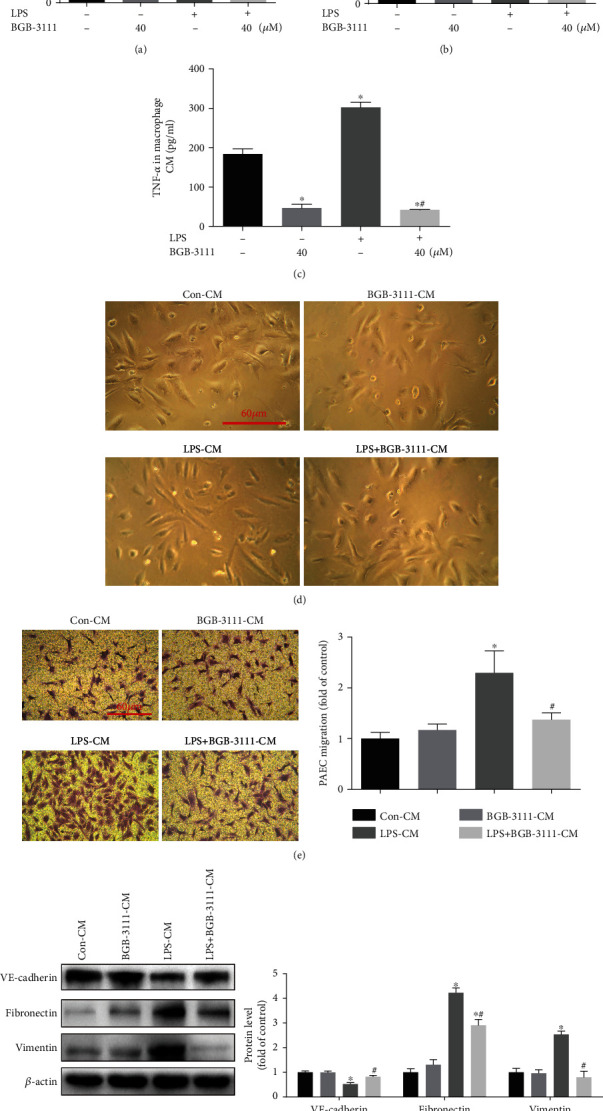
BTK inhibition protects endothelial-to-mesenchymal transition *in vitro* by a direct effect on macrophages. HPAECs were treated with different macrophage conditioned media. (a–c) Secretion of the inflammatory cytokines (IL-6, IL-1*β*, and TNF-*α*) in macrophage conditioned medium (CM) were determined via ELISA (*n* = 4–6). (d) Morphological examination was performed to detect morphology changes of HPAECs in each treatment group (*n* = 5). (e) Transwell migration assays were performed, and the number of migratory HPAECs was assessed by crystal violet staining (*n* = 5). TGF-*β*1+IL-1*β*+TNF-*α* served as a positive control. (f) Western blots were used to determine the expression of VE-cadherin, Fibronectin, and Vimentin in HPAECs (*n* = 3–4). Data are presented as mean ± SEM. ^∗^*P* < 0.05 vs. the Con-CM group; ^#^*P* < 0.05 vs. the LPS-CM group.

Abnormal immunity and inflammation appear to play a vital role in pulmonary endothelial dysfunction and vascular remodeling in PAH [2]. Previous studies reported that M1-polarized macrophages induced EndMT in infantile hemangiomas [29]. Meanwhile, EndMT-cells contribute to the pathogenesis of PAH [30]. To determine whether BTK mediated macrophage polarization affect EndMT in PAH, conditioned media (CM) from supernatants of macrophage cultures were collected to challenge cultured HPAECs. Analysis of macrophage CM by ELISA showed that BGB-3111 blocked the production of M1 macrophage-related proinflammatory cytokines (IL-6, IL-1*β*, and TNF-*α*) challenged by LPS (Figures 6(a)–6(c)). Next, we cultured HPAECs with different CM from macrophage cultures. Morphology assay revealed that HPAECs with cobblestone morphology transited to spindle-like morphology 48 h after LPS-CM challenge, which was partially inhibited by LPS+BGB-3111-CM (Figure 6(d)). Transwell migration assay showed that LPS-CM could significantly increase the migration of HPAECs compared to Con-CM, which was partially inhibited by LPS+BGB-3111-CM (Figure 6(e)). Protein levels of EndMT-related markers including VE-cadherin, Fibronectin, and Vimentin were determined by Western blot analysis. Compared with LPS-CM-treated HPAECs, LPS+BGB-3111-CM significantly reversed the downregulation of endothelial marker VE-cadherin and suppressed the upregulation of mesenchymal marker Fibronectin and Vimentin in HPAECs (Figure 6(f)). Together, these results demonstrated that M1-polarized macrophages could significantly promote the process of EndMT in HPAECs, which could be alleviated by BGB-3111.

## 4. Discussion

PAH is a progressive cardiopulmonary disorder characterized by pulmonary vascular remodeling and eventually leading to right heart failure and death. Crucial pathological changes in PAH include the disordered proliferation of endothelial cells, EndMT, smooth muscle cell proliferation, migration, and vasoconstriction, inflammatory cell infiltration, and extracellular matrix remodeling in the pulmonary arteries. Our data indicate that BTK plays a distinct role in the development of PAH. Pharmacological inhibition of BTK-alleviated MCT-induced PAH, pulmonary vascular remodeling, right ventricle hypertrophy, and macrophage-related inflammation in rats. BTKi modulated macrophage recruitment and M1 macrophage polarization, as well as the production of M1-related inflammatory cytokines both *in vivo* and *in vitro*. These therapeutic effects of BTKi might be attributed to the inhibition of NF-*κ*B/MAPK signaling pathways and the inhibition of NLRP3 inflammasome activation. Furthermore, BTKi reduced EndMT by inhibiting the production of inflammatory cytokines in macrophages. In summary, these findings suggest that BTK could be a potential target for the treatment of PAH by regulating macrophage recruitment and polarization.

BTK is a nonreceptor tyrosine kinase in the Tec family and is involved in B-cell development, Toll-like receptor triggering, and Fc receptor signaling [31]. Therefore, targeting BTK could be beneficial in different diseases characterized by pathologic antibodies, macrophage activation, and myeloid-derived type I interferon responses. Previously, it was reported that hypoxia induced accumulation of BTK^+^F4/80^+^ macrophages in the inflamed lungs [16]. Using double immunofluorescence staining, we found that BTK expression was upregulated in the lungs with MCT-induced PAH and especially colocalized with CD68^+^ macrophages. Thus, we suggested that BTK activation was involved in PAH pathogenesis by regulating macrophage-mediated inflammation. In our present study, we found that BGB-3111, a BTK inhibitor, prevented MCT-induced PAH and right heart hypertrophy. Data from both *in vivo* and *in vitro* studies suggest that the beneficial effects of BTKi on pulmonary hypertension and pulmonary vascular remodeling can be generally summarized into three aspects: (1) alleviating macrophage-related inflammation in the lung; (2) suppressing excessive M1 macrophage polarization, and (3) protecting endothelium against EndMT and preventing adventitia fibrosis.

Inflammation is a prominent pathological feature in pulmonary hypertension. Mounting evidence showed that altered immune mechanisms play a key role in the pathogenesis of PAH via recruitment of inflammatory cells, response of vascular cells to inflammatory stimuli, and autoimmune responses [4, 32]. Moreover, elevated proinflammatory cytokines and chemokines are crucial in the development of PAH by increasing vascular reactivity, inducing right ventricular hypertrophy, enhancing muscularization of the distal vessels, and mediating pulmonary vascular remodeling. Recently, several drugs that targeting on inflammation and immune dysfunction (for example, ubenimex, rituximab, tocilizumab, and anakinra) are under investigation [33]. Macrophages are key orchestrators of the inflammatory and repair responses in lung tissues and have been demonstrated to be vital in the initiation and progression of pulmonary hypertension [32, 34]. In hypoxia-induced pulmonary hypertension and portopulmonary hypertension models, macrophage depletion or inactivation prevents the development of PAH [7, 8]. These findings further indicate that suppression of macrophage-mediated inflammation could prevent the development of PAH. Up till now, the anti-inflammation effects of BTKi have been systematically investigated in diverse autoimmune diseases (including rheumatoid arthritis, Sjogren's syndrome, and type 1 diabetes) and other disease models, such as ischemia-reperfusion injury, asthma, and lung injury [13, 15, 35, 36]. In monocytes/macrophages, BTKi has been documented to inhibit the production of proinflammatory cytokines such as TNF-*α*, IL-1*β*, and IL-6 [37]. Moreover, BTK has been reported as a direct regulator of a key innate inflammatory machinery, the NLRP3 inflammasome in macrophages/neutrophils [38]. We hypothesized that BTK might involve the development of PAH by regulating macrophage-mediated inflammation. Our study is consistent with previous reports that MCT could lead to an upregulation of macrophage infiltration and inflammation in the lung. Pharmacological inhibition of BTK significantly inhibited MCT-induced accumulation of macrophages (including perivascular CD68^+^ macrophages and alveolar macrophages), as well as suppressed MCT-induced overexpression of M1-related inflammatory cytokines (IL-1*β*, IL-6, and TNF-*α*) in the lung. Collectively, these results suggest that BTKi effectively ameliorates MCT-induced PAH and pulmonary vascular remodeling by inhibiting macrophage-related inflammation, which eventually improves functional outcome in PAH.

Macrophages are a functionally heterogeneous cell population and can be polarized into classic inflammatory M1 and immunosuppressive M2 macrophages in response to diverse environmental changes. Recently, it was reported that macrophage M1/M2 polarization is critical for the development and progression of PAH [6]. Modulation of macrophage recruitment and phenotype via CX3CR1 inhibition can be a novel therapeutic approach for PAH [6]. However, little is known regarding whether BTK can regulate macrophage polarization in PAH. Previous studies reported that BTK regulates M1 macrophage differentiation and promotes the development of allergic inflammation [17]. Moreover, BTKi PCI-32765 treatment attenuated collagen and collagen antibody-induced macrophage activation in autoimmune arthritis and lowered the increase of M1-related inflammatory cytokines in the rat lung tissues [18]. Here, we found that in addition to inhibiting macrophage infiltration, BTKi also dampened M1 macrophage polarization and decreased the levels of M1-related inflammatory cytokines in rats with MCT-induced PAH. This result was further confirmed *in vitro* by using LPS-challenged U937-derived macrophages (a widely used model of M1 macrophage polarization). BTKi treatment not only suppressed LPS-induced M1 polarization of macrophages by inhibiting iNOS generation but also inhibited the production of M1-related inflammatory cytokines in macrophages. Together, these results suggest that BTKi attenuates MCT-induced inflammation via regulating macrophage activation and polarization in the lung.

Following stimulation of macrophages with LPS, IFN-*γ* or TNF*α*, M1 macrophage polarization is mediated by the activation of STAT1, p65 NF-*κ*B, PI3K, and MAPK, resulting in elevated production of inflammatory cytokines, chemokines, and iNOS. It was reported that BTKi ibrutinib could inhibit macrophage M1 polarization via suppressing the phosphorylation of ERK, AKT, and IĸB*α* in the presence or absence of LPS stimulation [17, 37]. Thus, NF-*κ*B and MAPK signaling pathways were examined to uncover the underlying mechanisms of BGB-3111 in LPS-induced M1 macrophage polarization. Our results showed that BGB-3111 blocked LPS-induced phosphorylation of ERK and p38 MAPK. In addition, BGB-3111 also reduced the phosphorylation of I*κ*B*α* and p65 NF-*κ*B in LPS-stimulated macrophages. We also showed that BGB-3111 inhibited MCT-induced NF-*κ*B and MAPK signaling pathways. Thus, these results suggest that either NF-*κ*B or MAPK signaling pathways are involved in BTK-mediated M1 macrophage polarization and pulmonary vascular remodeling.

NLRP3 inflammasome, a multiprotein complex involving the core sensor NLRP3, the adaptor apoptosis-associated speck-like protein containing the caspase-recruitment domain (ASC), and the proteolytic enzyme caspase-1, has recently emerged as a key molecular platform for inflammation. Studies have shown that the activation of the NLRP3 inflammasome and subsequent IL-1*β* production contributes to the inflammatory and remodeling process in PAH [39]. Blockade of the inflammasome adaptor ASC or upstream molecular P2X7 receptor attenuated the development and progression of pulmonary hypertension [40, 41]. Additionally, it was reported that luteolin inhibits macrophage polarization M1 phenotype via inhibiting NLRP3 inflammasomes activation in acute inflammation. Thus, therapeutic intervention targeting NLRP3 inflammasome in macrophages may be beneficial for PAH. Most recently, BTK was identified as a direct regulator in NLRP3 inflammasome activation [42]. In brain ischemia/reperfusion injury, BTK was required for NLRP3 inflammasome-dependent IL-1*β* processing in macrophages and BTK inhibition effectively impaired NLRP3 inflammasome activation and efficiently improved neurological function [15] In terms of mechanisms, BTK can physically interact with NLRP3 and ASC, resulting in the induction of ASC oligomerization and caspase-1 activation *in vitro* [15]. Therefore, NLRP3 inflammasome suppression was thought to be a potential mechanism for the protective effects of BTKi in PAH. In the present study, we found that BGB-3111 inhibited LPS-induced NLRP3 upregulation and caspase-1 activation and thereafter decreased IL-1*β* production in macrophages. Collectively, our data suggest that BTK inhibition could suppress M1 macrophage polarization and inflammation through inactivation of the NLRP3 inflammasome/caspase-1/IL-1*β* axis.

Altered pulmonary endothelial communication with immune cells is involved in the pathogenesis of PAH [2]. In pulmonary arterial hypertension, increased shear stress from increased flow, hypoxia, inflammation, and altered bone morphogenic protein receptor 2 (BMPR2) signaling can drive macrophage-mediated inflammation, thereby triggering extracellular matrix accumulation and smooth muscle cell proliferation in pulmonary vascular remodeling [4, 43, 44]. Moreover, M1-related inflammatory cytokines (IL-1*β*, IL-6, IFN-*γ*, and TNF-*α*) can promote the migration and EndMT of HPAECs, contributing to matrix deposition and pulmonary arterial remodeling [29]. This will also lead to increased inflammatory cell infiltration and proinflammatory cytokine production, which subsequently perpetuate inflammatory and autoimmune responses by macrophages, resulting in increased vascular wall thickness and pulmonary vascular remodeling [45, 46]. Nowadays, it is gradually accepted that EndMT participates in the development of PAH [47]. Generally, EndMT is characterized by the acquisition of mesenchymal cell markers (such as *α*-SMA, Vimentin, and Fibronectin) while the loss of endothelial cell markers (such as CD31 and VE-cadherin) in endothelium. In our current study, we also found a downregulation of VE-cadherin while an upregulation of *α*-SMA and Vimentin in MCT-induced PAH, which was reversed by the treatment of BGB-3111. Therefore, we further investigate the direct link between macrophages and EndMT and determine whether BTKi protects EndMT by a direct effect on macrophages. Our *in vitro* studies showed that M1 macrophages could release proinflammatory cytokines and promote EndMT, contributing to pulmonary vascular remodeling. However, BTKi effectively downregulated IL-6, IL-1*β*, and TNF-*α* production in macrophage CM which inhibited the migration and EndMT of HPAECs. Therefore, these data from either *in vivo* or *in vitro* studies suggest that BTKi might inhibit M1 macrophage polarization and inflammation, subsequently rescuing EndMT and collagen deposition in PAH. BTKi protects EndMT by a direct effect on macrophages.

## 5. Conclusions

**Figure 7:**
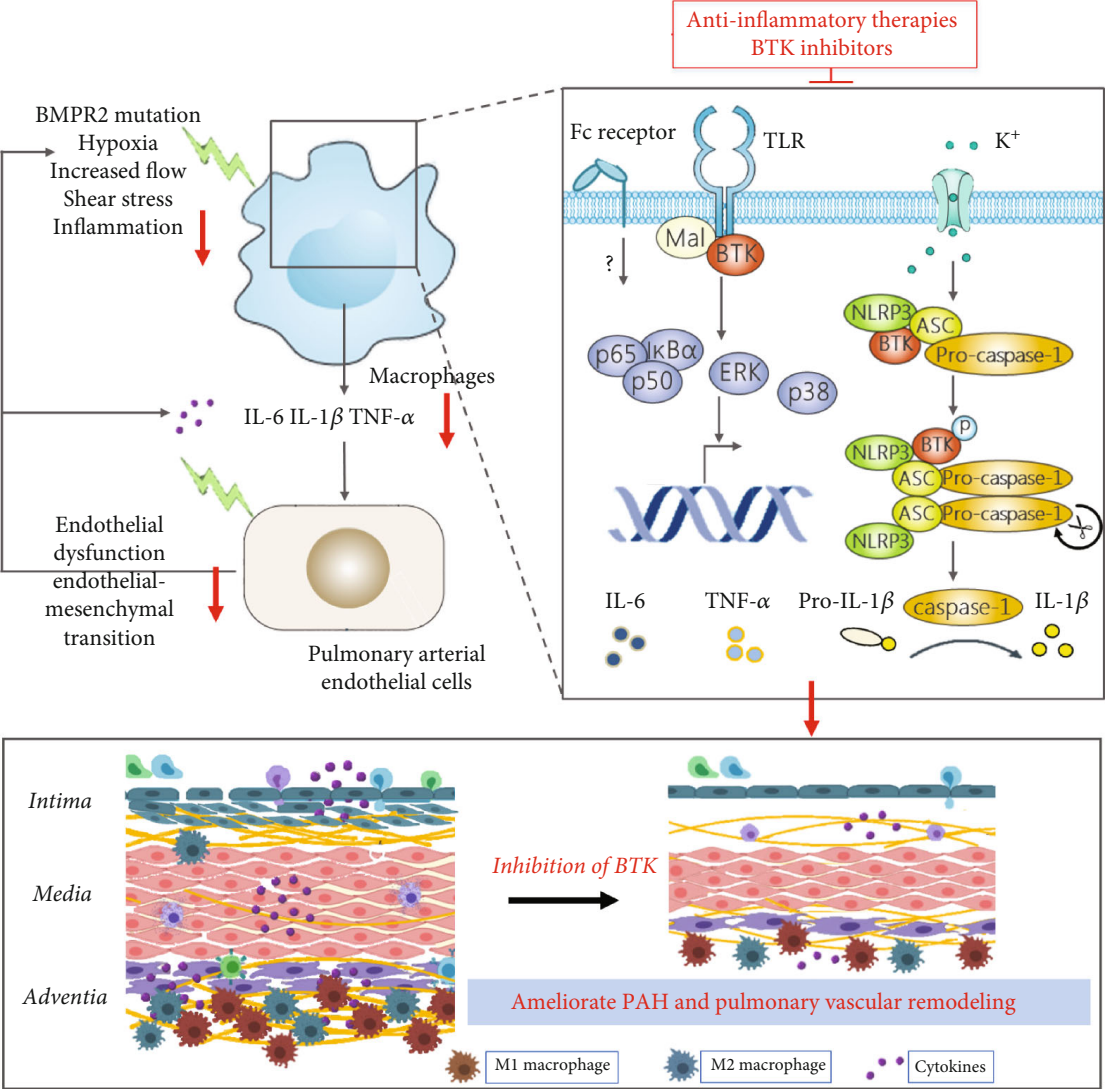
A proposed model illustrating the therapeutic effect of BTK inhibitor on PAH. Inhibition of BTK suppressed NLRP3 inflammasome/caspase-1/IL-1*β* axis and NF-*κ*B/MAPK signaling and then reduced the production of M1-related inflammatory cytokines (IL-6, IL-1*β*, and TNF-*α*) in macrophages. BTK inhibitor might inhibit M1 macrophage polarization and macrophage-related inflammation, subsequently rescuing EndMT and collagen deposition, and then ameliorate MCT-induced PAH and pulmonary vascular remodeling. Inhibition of BTK ameliorates PAH by modulating macrophage polarization and inflammation.

In conclusion, the present study demonstrated that BGB-3111, a selective BTK inhibitor, can ameliorate MCT-induced PAH and pulmonary vascular remodeling. These beneficial effects of BTK inhibition are associated with the suppression of NLRP3 inflammasome and NF-*κ*B/MAPK signaling, thereafter reduce M1 macrophage polarization and M1-related inflammatory cytokine production, and thereafter, alleviate EndMT and pulmonary vascular remodeling (Figure 7). Further studies are required to evaluate the therapeutic potential of BTKi in other pulmonary hypertension models. Taken together, these findings suggested that BTK might be a novel therapeutic target for the treatment of PAH.

## Figures and Tables

**Table 1:** The primer sequences of targeted RNA.

Gene primer	Species		Sequence (5′ to 3′)
*BTK*	Rat	Forward	TATGAAGGAACTGCTTTGACTC
		Reverse	TAATGGCTGCCTCTTGAACTA
*β-Actin*	Rat	Forward	CTGAACCCTAAGGCCAACCG
		Reverse	GACCAGAGGCATACAGGGACAA
*IL-6*	Human	Forward	CCTCCAGAACAGATTTGAGAGTAGT
		Reverse	GGGTCAGGGGTGGTTATTGC
*IL-1β*	Human	Forward	TGAAATGATGGCTTATTACAGTGGC
		Reverse	GTAGTGGTGGTCGGAGATTCGTAG
*TNF-α*	Human	Forward	AAGCAACAAGACCACCACTTCGA
		Reverse	AGATTCCAGATGTCAGGGATCAAA
*iNOS*	Human	Forward	GAGCATCACCCCCGTGTTTCA
		Reverse	TCTTGGGTCTCCGCTTCTCGTC
*Arg-1*	Human	Forward	TAACTCGAACAGTGAACACAGCAG
		Reverse	TAGGTGGGTTAAGGTAGTCAATAGG
*β-Actin*	Human	Forward	TGAGACCTTCAACACCCCAGC
		Reverse	ACAGCTTCTCCTTAATGTCACGC

BTK: Bruton's tyrosine kinase; IL-6: interleukin-6; IL-1*β*: interleukin-1*β*; TNF-*α*: tumor necrosis factor-*α*; iNOS: inducible nitric oxide synthase; Arg-1: arginase-1.

## Data Availability

The data used to support the findings of this study are available from the corresponding author upon request.

## Supplementary Materials

Supplementary Materials Supplementary Figure 1: representative images and analysis of blotting for BTK in rat lung tissues. Supplementary Figure 2: representative images and analysis of blotting for p65 NF-*κ*B, ERK, and p38 MAPK in rat lung tissues.
