# Supplementary material for: Inhibition of Bruton's Tyrosine Kinase Alleviates Monocrotaline-Induced Pulmonary Arterial Hypertension by Modulating Macrophage Polarization
Source: Oxid Med Cell Longev. 2022 Aug 29;2022:6526036. doi: 10.1155/2022/6526036 (PMC9444460; doi:10.1155/2022/6526036)
Supplement: Supplementary Materials — Supplementary Figure 1: representative images and analysis of blotting for BTK in rat lung tissues. Supplementary Figure 2: representative images and analysis of blotting for p65 NF-κB, ERK, and p38 MAPK in rat lung tissues. [file 6526036.f1.docx]

**Supplementary Figure 1**


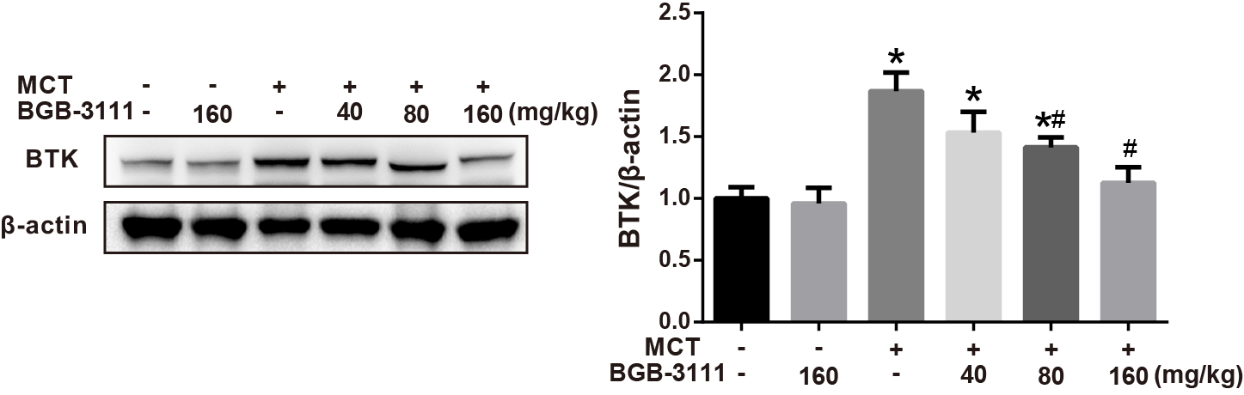


Supplementary Figure 1: Representative images and analysis of blotting for BTK in rat lung tissues. Data are presented as mean±SEM, n=5. ^∗^*P*<0.05 vs control; ^#^*P* < 0.05 vs MCT.

**Supplementary Figure 2**


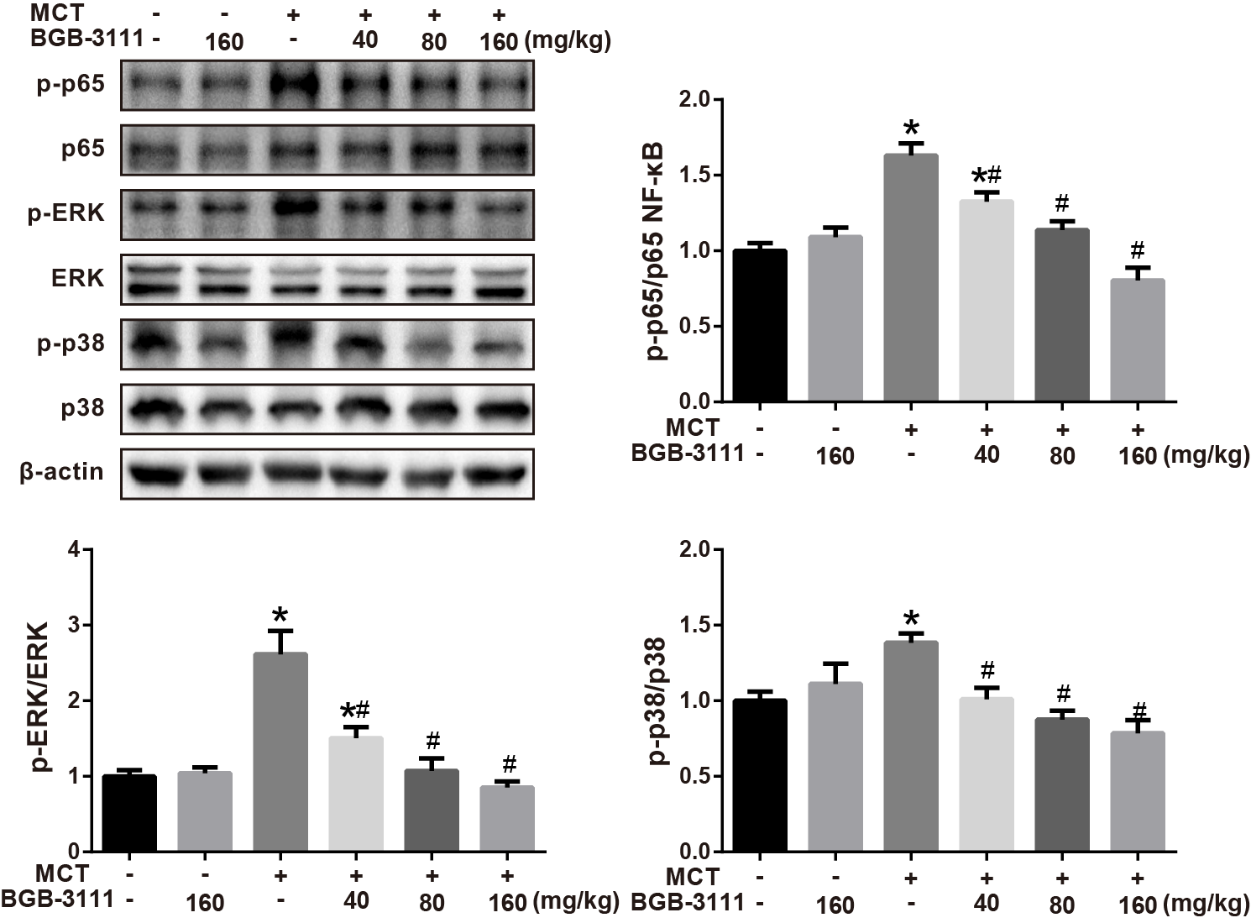


Supplementary Figure 2: Representative images and analysis of blotting for p65 NF-κB, ERK and p38 MAPK in rat lung tissues. Data are presented as mean±SEM, n=4-6. ^∗^*P*<0.05 vs control; ^#^*P* < 0.05 vs MCT.
